# DMC reports in the 21st century: towards better tools for decision-making

**DOI:** 10.1186/s13063-023-07290-4

**Published:** 2023-04-21

**Authors:** Marc Vandemeulebroecke, Mark Baillie, Ardalan Mirshani, Emmanuel Lesaffre

**Affiliations:** 1 Novartis Pharma AG, Basel, Switzerland; 2 Novartis Institutes of Biomedical Research, Cambridge, USA; 3 L-Biostat, KU Leuven, Leuven, Belgium

**Keywords:** Data Monitoring Committee, Data Safety Monitoring Board, DMC report; Good graphical principles, Statistical graphics

## Abstract

Data Monitoring Committees (DMCs) have the important task to protect the safety of current and future patients during the conduct of a clinical study. Unfortunately, their work is often made difficult by voluminous DMC reports that are poorly structured and difficult to digest. In this article, we suggest improved solutions. Starting from a principled approach and building upon previous proposals, we offer concrete and easily understood displays, including related computer code. While leveraging modern tools, the most important is that these displays support the DMC’s workflow in answering the relevant questions of interest. We hope that the adoption of these proposals can ease the task of DMCs, and importantly, lead to better decision-making for the benefit of patients.

**Supplementary Information:**

The online version contains supplementary material available at 10.1186/s13063-023-07290-4.

## Introduction

Independent Data Monitoring Committees (DMCs), also called Data Safety Monitoring Boards, have the task to protect the safety of current and future patients during the conduct of a clinical study, usually in phase 2 or phase 3 studies. Regulatory agencies have developed guidelines for the DMC process, see, e.g., the European Medicines Agency Guideline on DMCs [1] and the US Food and Drug Administration Guidance on Establishment and Operation of Clinical Trial DMCs [2]. Here, we focus on the construction of the DMC report.

Typically, DMCs are composed of a small number of clinicians and one statistician. They are tasked with the review of interim reports that describe adverse events and other data relevant for the assessment of patient safety during the conduct of the study, as well as, if needed, efficacy data for the assessment of benefit-risk. As patients are continued to be recruited and treated in the study, this assessment often must be done within a few days once the report has been received by the DMC. In addition, each DMC member has only a few hours allotted to this task per the contractual agreement with the study sponsor. It is, however, not uncommon that the DMC is confronted with voluminous reports that are poorly structured and difficult to digest. To quote only some of many authors:

- Buhr et al. [3] note that “many reports are unclear and unfocused.”
- DeMets and Wittes [4] state that “too many Data Monitoring Committee Reports for interim review of trial progress are quite inadequate for Data Monitoring Committees to make informed decisions about risks and benefits.”
- Wang et al. [5] assert that “the review of clinical safety data can be tedious and time consuming. Reviewers can feel like they are looking for a needle in a haystack.”
- Zink et al. [6] stress that “analysis and interpretation are made more difficult by the sheer number and variety of events that occur.”
- Wildfire et al. [7] complain about “many pages of static displays.”
- O’Connell and Pietzko [8] highlight “short available time windows”, and that “paging through tables is inefficient.”
- Evans et al. [9] quote NEJM Evidence Editor Dr. Jeff Drazen, calling DMC service the “toughest job in clinical trials.”

Our experience is in line with these points. Despite today’s computer power and capabilities of producing magnificent graphical displays, most DMC reports are based on printouts that resemble much of the line printer outputs of the 1970–1980s. Looking at such old-fashioned tables and listings is tiring and one quickly gets lost in the details. Figures are often of poor quality or not even included at all. Such DMC reports can amount to more than 1000 pages of output per interim look for a single study. Considering that there are often multiple DMC analyses per study, increasingly complex study designs (adaptive designs, platform trials, etc.), and often multiple related studies combined in the same review, the task of the DMC appears daunting.

It is surprising to see that the DMC reports often lack clarity, because the stakes are high. Missing an important safety issue can put the health of current and future patients at risk. The trial sponsor may incur reputational damage, waste resources on a development program without any future, or — for safety signals that can be addressed or mitigated — may take necessary actions too late. This can imply substantial extra costs for the company later, or possibly even result in withdrawing the drug from the market.

What are the reasons for this grievance? Do we not have appropriate tools, or have we not put enough thought in the construction of appropriate processes and data displays? A wealth of knowledge does exist. For example, Fleming et al. [10] discuss best practices and operating principles for DMCs. They also comment on creating the DMC charter and the DMC report; see also Fleming et al. [11] and Calis et al. [12]. Buhr et al. [3] discuss which organizational setup, report structure, and graph types are most helpful for facilitating a well-informed DMC review. Wildfire et al. [7] introduce an interactive safety explorer suite which allows the user to quickly switch between various displays, covering and linking group-level as well as individual-level information. Wang et al. [5] offer concrete guidance on an appropriate choice of displays for an efficient reviewer workflow. Mütze and Friede [13] give an account of an interactive DMC experience in a COVID-19 trial. Thomas et al. [14] and Zink et al. [6] propose further useful plots. Duke et al. [15] and Vandemeulebroecke et al. [16] provide guidance on good graphical principles; the CTSpedia compendium of graphs [17] (with code) is related to the former. Why is this wealth of knowledge and material not taken up and routinely implemented?

We posit that part of the reason is lack of awareness, but also system inertia and a flawed cost or incentive system. Sponsors want to “cover all bases” and maximize the material that is provided to the DMC, instead of making it easily digestible. Producers of DMC reports often lack the experience of having served on a DMC. In addition, many technical solutions for producing DMC reports are not putting the clients and their questions and workflows front and center. With this article, we want to increase awareness, synthetize existing proposals, and suggest possible solutions. We primarily consider industry-sponsored trials, but similar principles apply also more broadly. Starting from a principled approach, we offer concrete solutions including corresponding computer code in R [18], using publicly available data from a clinical trial [19]. In doing so, our principle is that we must support the DMC’s workflow in answering the questions of interest to them. It does not suffice to introduce more sophisticated graphical tools and technical solutions. Only those solutions that are easily digestible and specifically designed to answer the questions which are relevant to the DMC should be considered. A systematic implementation of these ideas may require some effort initially, compared to simply continuing with current practice. However, we believe that these efforts will pay off quickly, both financially and in terms of risk minimization. Importantly, they will lead to better decision-making for the benefit of patients.

The rest of this paper is structured as follows. Section 2 illustrates the challenges that DMCs are nowadays confronted with, then specifies the questions a DMC most importantly wants to address, and on how best to approach this task. In Section 3, we propose a purposeful structure for a DMC report. In Section 4, we zoom in on some of the most commonly produced DMC outputs, and we provide tailored and effective alternatives. Section 5 briefly comments on efficacy and benefit-risk assessments. Sections 6 and 7 close with a discussion and a short conclusion, respectively. Source tables are included in an additional file. We do not provide a one-size-fits-all solution. Rather, our intention is to provide a useful starting point for designing a DMC report which, of course, should also take the DMC members’ input into account.

## DMC reports: seeking the needle in a haystack?

A typical safety DMC report for a randomized clinical trial includes tables that compare the baseline characteristics between the treatment arms, such as the number of patients screened and screen failures, demographics, medical history, together with prior therapies and concomitant medications. Key is the overall table of adverse events followed by adverse event tables split by system organ class, preferred term, severity, and relationship to treatment. Additional tables follow of serious adverse events or those that lead to treatment discontinuation. Next to these, other tables are produced that contrast the laboratory parameters between the treatment arms and at each time of examination. Comparisons with baseline values are also standardly included, using, e.g., shift tables describing any transitions of laboratory values to in- or outside the normal range. This is also done for vital signs such as blood pressure and body temperature. Figures may not be provided at all. Listings of all numerical values used in the tables are usually given in a separate document.

It is then no surprise that for a study of a few hundred patients, a DMC report easily ends up in more than 1000 pages. The information given is more than sufficient to do a proper evaluation of the safety (and if applicable also the efficacy) of the patients. The problem is that the information is rather disparate. For instance, switching from the overall to the individual level is typically difficult. The information on, say, a laboratory parameter as summarized in a table is often not connected to the corresponding figure (if available) or to individual listings. A report often does not even contain a table of contents with page numbers to help with locating related information. Therefore, relating one parameter to another is not easy, even though this can be informative to assess patterns of clinical interest. The simple yet important question of “what has changed since the previous DMC” is equally difficult to answer. These examples illustrate what we believe is the core of the problem: the typical DMC reports are not specifically designed to support the DMC members’ workflow in addressing their questions of interest. Rather, they are often simply organized according to the way the data are recorded in the database, and/or taken as a subset of the outputs planned for the Clinical Study Report.

Surely, there is an element of format and visual appearance, when DMC reports are voluminous collections of tables and listings. Amit et al. [20] note that the risk of missing an important safety issue is lower with appropriate graphs than with routinely produced tables. In addition, “well-designed graphs improve communication between statisticians and clinicians.” Emphasis should be put on the qualifiers “appropriate” and “well-designed”. We will gain nothing if we replace large collections of tables by difficult-to-navigate collections of graphs, or if we provide so many interactive options that it overwhelms the DMC. Finding the needle in a haystack does not become easier by adding more hay. We refer to Gordon and Finch [21] for a sobering assessment of current practice in scientific graphs. On the topic of interactivity, we quote Wang et al. [5] who “have observed some visual designers getting carried away with packages that contain many interactivities and features, which left reviewers overwhelmed.” Novelty, versatility, or visual appeal do not help if they are not tailored towards the task at hand. So, what can we do to help the DMC members complete their task more efficiently?

In essence, we need to solve the problem of *facilitating information-seeking while avoiding information overload*. In his visionary essay “As we may think”, Bush [22] spells out the challenges with too much information and the need to devise efficient mechanisms to control and channel information for effectively answering questions of interest. The need to channel an exploding amount of information towards its effective use for a particular task has all but disappeared. This is precisely what DMCs are facing today, with or without modern tools and software. To quote Buhr et al., [3] there is “so much information in so disorganized a manner that the DMC is overwhelmed with unnecessary and irrelevant detail […]. The DMC report must facilitate efficient review of comprehensive data through a well-designed report structure and thoughtful organization of analyses”.

In other words, we need to put the “end-user (reviewer) needs first” [23]. We need to recognize that the DMC members work *collaboratively* and *iteratively* in identifying patterns and seeking information on potentially emerging safety signals. The DMC package and process should be specifically designed to support this way of working, in line with Shneiderman’s so-called information-seeking mantra: [24] “overview first, zoom and filter, then details-on-demand”. Most importantly, we need to spell out the specific questions a DMC is typically interested in addressing and start from these questions [25].

In our experience, in an ongoing late-phase randomized clinical trial, the DMC typically focuses on the following questions:

- *Is there an imbalance* in any relevant safety aspect?
- If so, *can it be explained from an imbalance at baseline*?
- And *does it show meaningful relations across parameters and/or domains*?
- *In the presence of a safety imbalance, what is the benefit-risk profile of the experimental treatment?*

Of special interest are so-called “*outliers*,” as well as the element of *time*. For example, how long did an abnormality persist? Did its timing relate to other abnormalities and/or to treatment, concomitant medications etc.?

How do DMC members typically approach the task of addressing these questions? The starting point is usually some type of central overview at the summary level (by treatment arm). Then, DMC members will move back and forth investigating notable points or questions. In particular, they will oscillate:

- Between group level and individual level,
- Between post-treatment and pre-treatment data,
- Across parameters and domains, and
- Potentially between safety and efficacy information to assess the benefit-risk tradeoff.

Any set of material that is organized and designed to address these questions of interest, to support this workflow of how to investigate them, and to allow doing all this quickly, is good material for the DMC.

## A purposeful structure of a DMC report

A purposeful DMC report should assemble the relevant tables and figures in a way that supports the DMC’s decision process. Only data relevant to the DMC should be displayed. Below we suggest a structure which we consider useful, while acknowledging that other structures might be chosen depending on the characteristics of the study (or studies) at hand. As a useful default, we imagine the DMC report as a single, clearly structured, and continuously page-numbered document, including relevant internal cross-references or hyperlinks. Interactive versions or supplements as proposed by Wildfire et al. [7] and Wang et al., [5] if available, can help for data exploration, including the functionality to obtain individual-level information when “hovering over” selected data points on a summary plot. But they should be created with restraint, a clear structure and good judgment. The structure suggested below can serve as a good starting point to explore the safety of the patients. The choice of the adverse events to inspect, as well as which laboratory parameters, vital signs, etc. to inspect should result from an interaction between the DMC clinicians, the DMC statistician, and the clinicians and statisticians of the sponsor.

As in Fleming et al., [10] we suggest to start the DMC report with”a brief protocol synopsis, a listing of new amendments, a reminder of previous recommendations of the DMC.” We also suggest to summarize major safety imbalances found in the previous DMC, which helps in evaluating what has changed since the last DMC meeting. Further, we believe that the following structure helps the DMC in reaching efficiently a justified recommendation to the sponsor.

1. Classically one starts with a baseline comparison of demographics, clinical history, concomitant medication, and operational characteristics of the trial (e.g., protocol violations and randomization details if stratification has been used). The tables of demographics should be replaced by graphical output as much as possible (see Fig. 1 below). Tables that enumerate all possible medical histories or concomitant medications can overwhelm the reader and are usually only quickly glanced over. It is better to select a relevant subset of clinical and treatment histories.

**Fig. 1:**
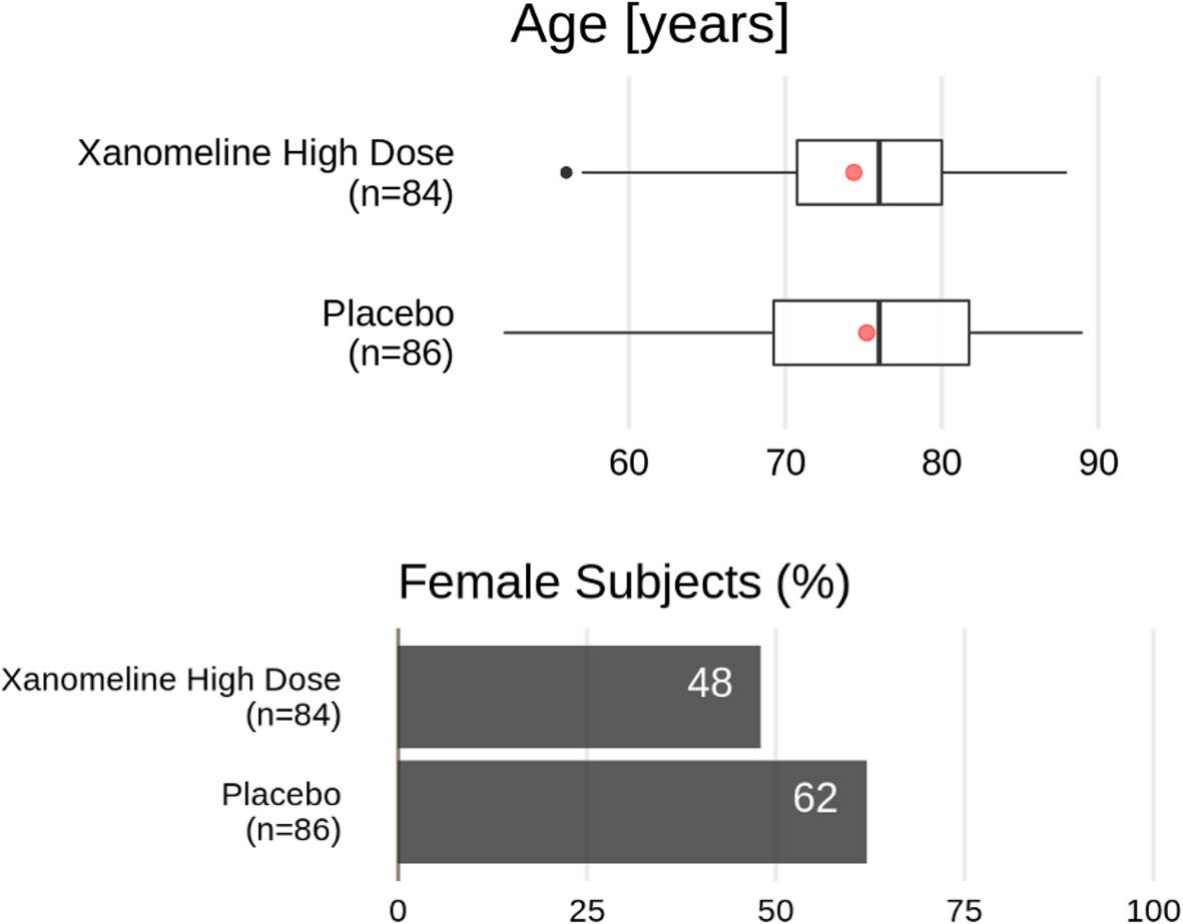
Demographics from the Xanomeline study (alternative). Top: Box-whisker plot with a red dot representing the mean of the data, the vertical line is located at the median of the data. The box covers the second and third quartile, while the whiskers extend to the last data point within 1.5 times the interquartile range. Bottom: bar plot of the percentage of female subjects in the respective treatment groups
2. Then should follow a comparison of the adverse events and serious adverse events, exposure-adjusted or not, starting with a bird’s eye view per treatment arm (as in Figs. 2 and/or 3 below). The outputs in point 1 could be consulted if there is a safety imbalance. A detailed clinical report of all patients with Common Terminology Criteria grade ≥ 3 events can follow immediately or could be put after the evaluation of the laboratory.

**Fig. 2:**
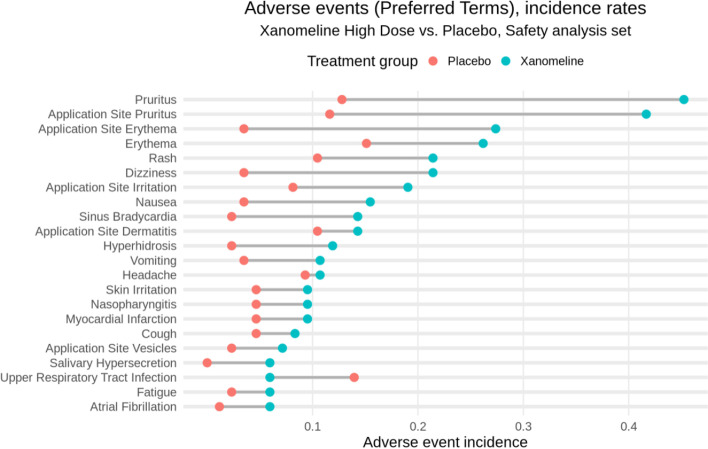
Adverse event overview for the Xanomeline study

**Fig. 3:**
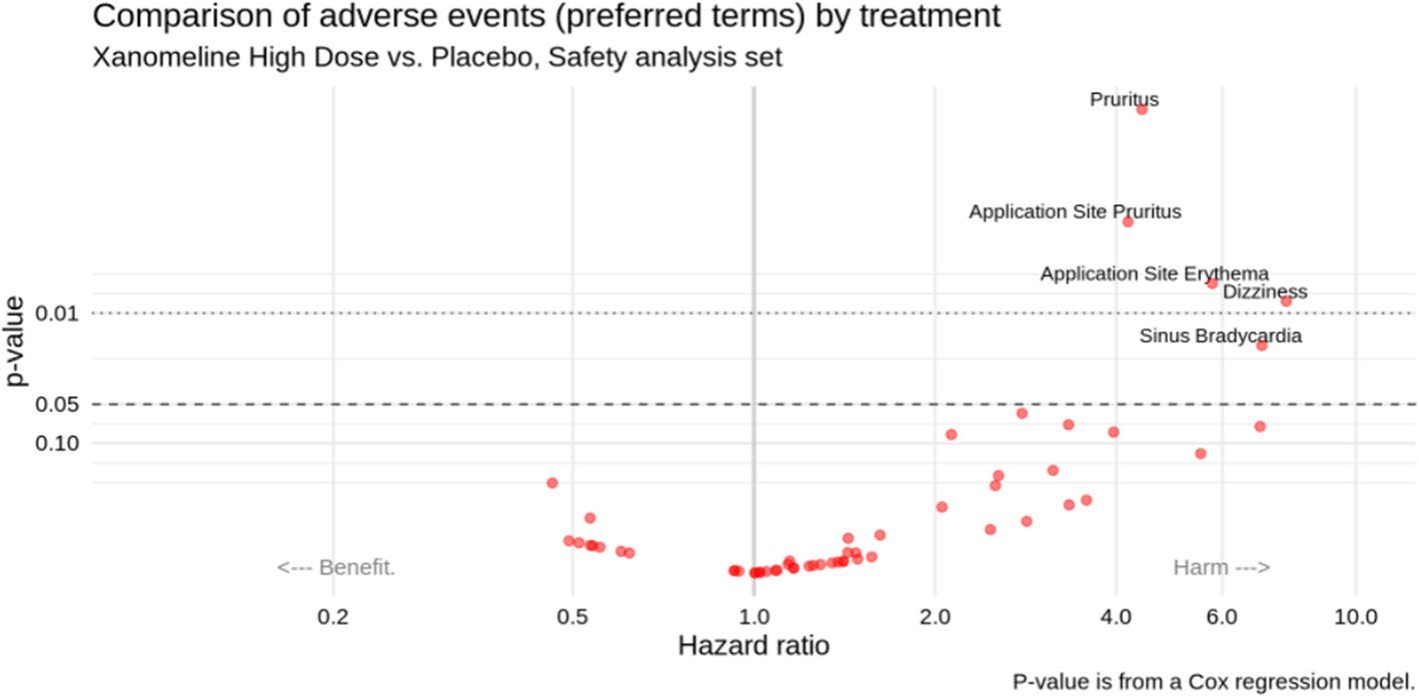
Adverse event “volcano plot” for the Xanomeline study (Xanomeline High dose vs. Placebo)
3. For the laboratory parameters, again it helps to provide a bird’s eye view first, followed by more details on individuals of interest. Figures 5 and 6 (see next section) together with spaghetti plots for patients with Common Terminology Criteria grade ≥ 3 values provide a good and sufficiently detailed comparison of the treatment arms.
4. The same as in point 3 can be done for vital signs.
5. Additional detailed tables, such as on adverse events or serious adverse events for the different analysis populations and on concomitant medication, can be put in an appendix.

## Examples of more meaningful DMC displays

To illustrate our points more concretely, we now collate useful graphical displays that address the most common questions of interest for a DMC. We start from what can be considered a typical set of outputs, which we take from a publicly available Clinical Study Report. Concretely, we harvest our source outputs from the Xanomeline Clinical Study Report which has been published, along with complete data, as part of the Clinical Data Interchange Standards Consortium efforts [19]. We acknowledge that more efficient actual DMC reports exist [13, 26], but they remain the exception thus far, and to our knowledge the corresponding data is not publicly available. We therefore believe that our illustrative approach based on the Xanomeline Clinical Study Report represents a realistic scenario in common practice, and it allows us to share code for our proposed solutions (see supplementary material).

In the following illustrations, we focus on three domains of interest: the study population; adverse events, and laboratory data. Other domains, such as vital signs, can be addressed analogously. The source outputs from the Xanomeline Clinical Study Report are in the supplementary material; the proposed alternatives are displayed below. For simplicity, we only show one of the two Xanomeline doses in the proposed plots.

### Study population

Supplementary Table 1 shows the baseline characteristics of the Xanomeline study population as taken from its Clinical Study Report. This type of table is very common in both Clinical Study Reports and DMC reports; it is clearly structured and relatively easy to digest since readers are used to it. However, it requires some effort to search for the most relevant comparisons within the wealth of numbers displayed. A graphical alternative is shown in Fig. 1. In this display, any systematic differences, as well as outliers in the continuous variables, “jump out” to the eye effortlessly. Essential information on key graphical elements is provided in the caption. Note that for a binary variable like sex, we only display one of the categories. We also omit other non-essential data, and certainly any p-values, since baseline testing for differences is considered bad practice [27].

### Adverse events

Supplementary Tables 2 and 3 summarize the incidences of adverse events and serious adverse events, respectively, in the Xanomeline study. Again, these two tables are highly common, and readers are used to scan them for any signals. However, from numbers alone, it is hard to get an intuitive understanding of any differences, and the adverse event table is too voluminous to be easily digestible. In practice, we distinguish between adverse events of special interest and any other adverse event. The number of adverse events of special interest is often small and inspired by findings found in Phase II studies or in past trials of similar drugs. In this case, a table such as Supplementary Table 3 is appropriate. For the other adverse events, we argue that graphical displays are more appropriate.

Many alternatives have been proposed for displaying adverse events. We highlight two options that we consider particularly effective. The first is a dotplot over Preferred Terms as shown in Fig. 2, in this case, ordered by the rates in the Xanomeline group. The plot is simple and intuitive, making any imbalances immediately clear. Wildfire et al. [7] have implemented this idea in an interactive system and expanded the graph into a “grable” (that is, a mixture of a graph and a table) to include numerical details (static implementations have also been proposed [20, 28]). In this graph, the display is grouped by System Organ Class with the option to expand the Preferred Terms within System Organ Class, whereby filters for seriousness, severity, relatedness to drug, and outcome can be applied. Wang et al. [5] provided a similar display. Finally, Fig. 2 can be adapted such that the time at risk is taken into account.

The second useful display is a “volcano plot” like in Fig. 3, showing the preferred terms in a scatter plot of *p*-values (or alternatively, false discovery rate adjusted *p*-values) against hazard ratios (or alternatively, risk differences) for two treatment groups (here: Xanomeline High dose vs. Placebo). Such a plot can be produced for any pair of treatment groups that are compared. This plot gives an immediate impression of the overall balance of risk (across all adverse events), as well as identifying the most unbalanced events. We used *p* < 0.05 as cutoff value to label the dots, but the developed R routines allow choosing other cutoff values for the *p*-values or false discovery rate adjusted *p*-values. (Alternatively, one can choose to label, say, the 10 most extreme adverse events.) Zink et al. [6] had proposed this display, along with additional features such as varying the dot size (by number of events) and color (by relative risk). This display was also implemented by Wang et al. [5] A simple uncluttered version as in Fig. 3 will already be very useful to a DMC. The nominal *p*-values in this figure should be interpreted as descriptive rather than inferential measures.

### Laboratory data

Clinical laboratory is a particularly difficult area to review. Many continuous and categorical variables are measured multiple times in various domains such as chemistry, hematology, and urinalysis. The interpretation of these many parameters requires specialized knowledge and should be put into context with other measures, especially adverse events. DMC outputs for laboratory data easily have the size of a small book, and the detection and judgment of putative safety signals in this wealth of numbers is difficult and often subjective. Supplementary Tables 4 to 6 show three of the most common outputs for laboratory: descriptive statistics of continuous variables (over time); frequencies of abnormal values (any time during treatment); and so-called “shift tables” (over time), that is, series of cross-tabulations of normal/abnormal values at baseline and post baseline. These three outputs alone sum up to 50 pages in the Xanomeline Clinical Study Report.

**Fig. 4:**
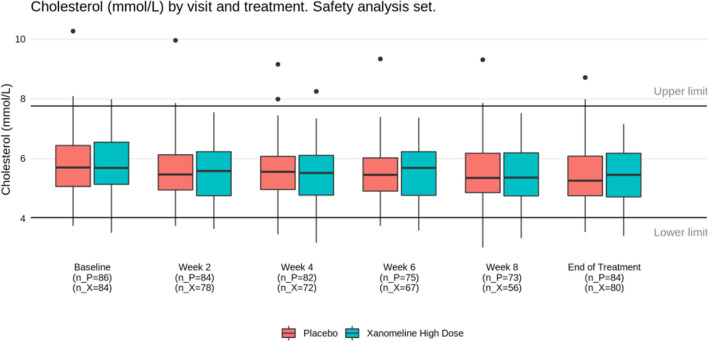
Laboratory parameter distribution over time from the Xanomeline study (here: cholesterol). Box-whisker plot with horizontal line representing the median of the data. The box covers the second and third quartile, while the whiskers extend to the last data point within 1.5 times the interquartile range. n_P, number of patients on Placebo; n_X, number of patients on Xanomeline High Dose

**Fig. 5:**
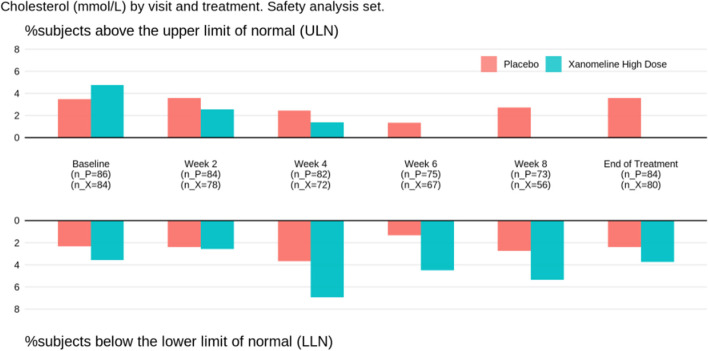
Abnormal laboratory parameter values over time from the Xanomeline study (here: cholesterol). n_P, number of patients on Placebo; n_X, number of patients on Xanomeline High Dose

Several alternatives have been proposed for a more effective display of the laboratory data, in particular by means of line plots (such as spaghetti plots) or plots of distributions (such as box plots or violin plots), sometimes including ancillary information and/or interactive features. We refer to the work by Harrell [29] and by the company Rho [30]. In our experience, and given the limited time (and sometimes limited technical savvy) of DMC members, simple yet effective displays are the best place to start — even if they omit some detailed information. We therefore recommend displays such as Figs. 4 and 5, possibly on a logarithmic scale. The former provides a comparison of distributional trends and outliers; the latter focuses on abnormal values (both over time). Both are easily understood at first glance.

**Fig. 6:**
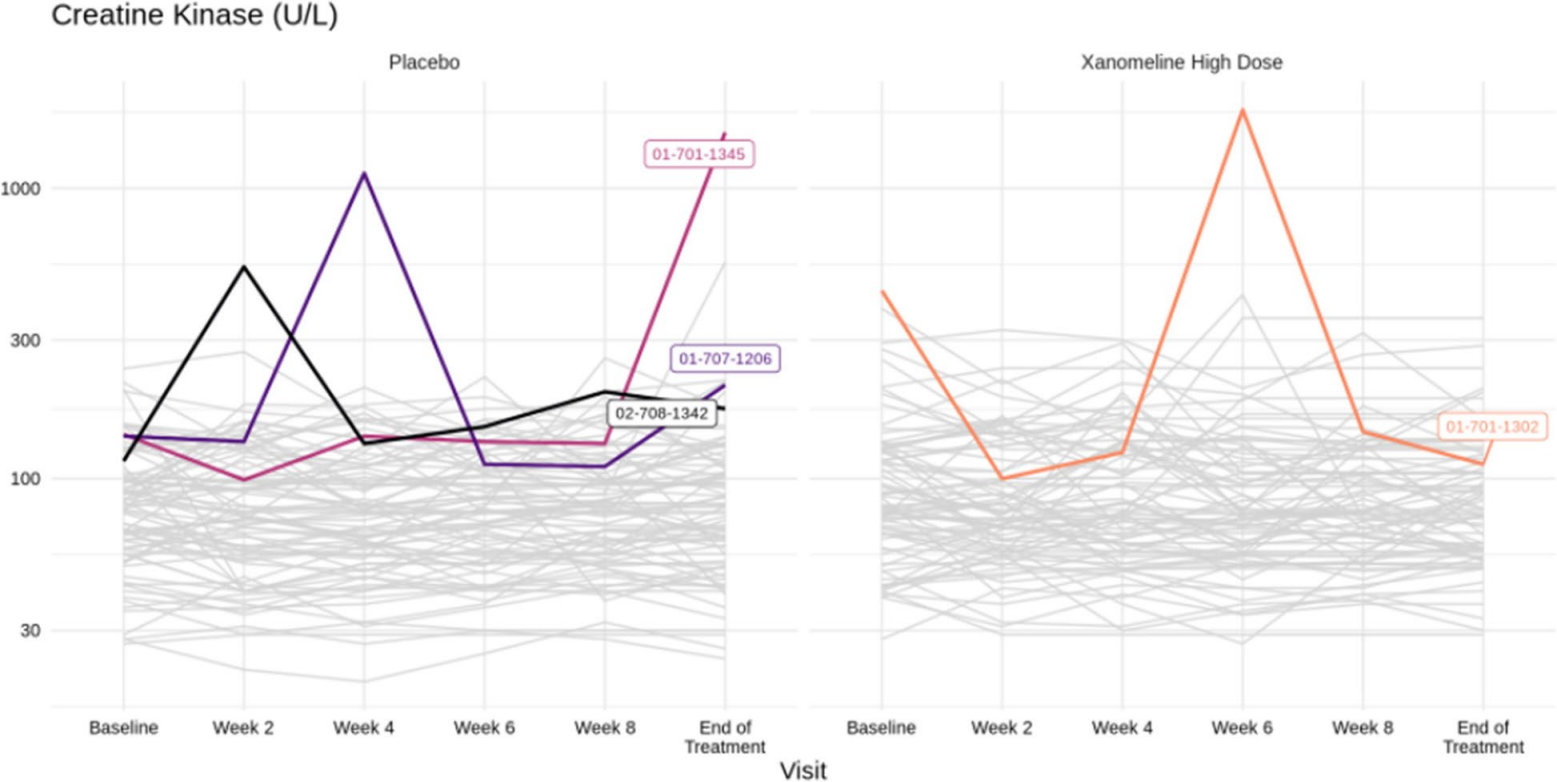
Spaghetti plot of creatinine kinase over time. Trajectories that exceed 3 times the upper limit of normal are colored, with annotated subject numbers. Horizontal reference lines can be toggled on for limits of normal if they are the same for every subject (which is not the case for this parameter)

Next to Figs. 4 and 5, there is a need to inspect individual profiles of the laboratory parameters over time for those subjects in whom the respective value has exceeded the normal range at least once, especially if the value reached Common Terminology Criteria grade ≥ 3. A line plot (spaghetti plot) can then indicate whether and/or how fast the value goes back to normal. An example is given in Fig. 6, including all other patients in gray to provide context. It can also be useful to put the laboratory parameter into context with the trajectories of other parameters in the same subject (using “small multiple” plots). Interactive applications can allow a flexible choice of parameters and/or subjects to display, see, e.g., the implementation by Rho [31].

**Fig. 7:**
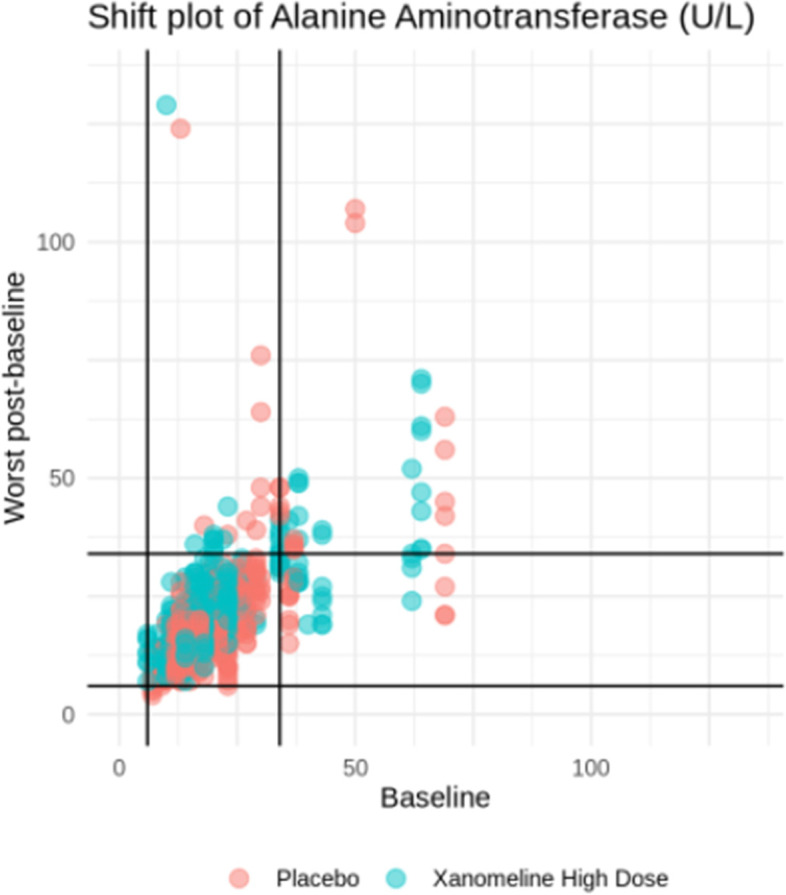
A graphical alternative to a laboratory shift table. Black lines are limits of normal

Sometimes shift tables are provided, which indicate the number of subjects that switch Common Terminology Criteria grade from baseline. However, in our experience, such tables are not easy to read. Better is to use a scatter plot such as Fig. 7. But we doubt whether even this provides useful additional information on top of the above-discussed profile (i.e., line) plots.

### Detailed clinical profiles of patients of special interest

**Fig. 8:**
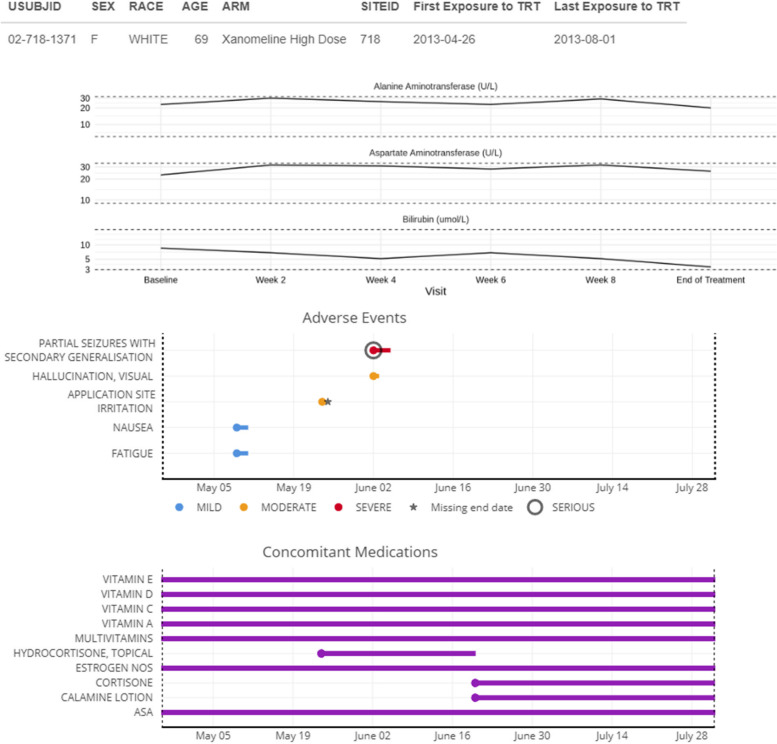
Patient profile. Description of the most relevant clinical information at a glance

Finally, clinicians need a complete medical profile of selected patients of special interest, particularly those that showed a serious adverse event and/or a laboratory value of, say, Common Terminology grade ≥ 3. A one-page summary of the patient’s demographics, medical history, concomitant treatment, current treatment, adverse events, and laboratory values will allow judging the clinical relevance of the adverse event or abnormal values. Such a summary also allows to appropriately examine rare serious adverse events. Figure 8 provides an example, including a graphical summary of key events over time. It can be further tailored or expanded towards the particular needs of the study at hand. We also refer to Powsner and Tufte [32] for an early proposal for a “graphical summary of patient status,” and to the recent patientProfilesVis implementation in R [33].

## Efficacy and benefit-risk assessment

In case of an imbalance in safety, the DMC evaluation depends not only on safety but on the benefit-risk profile of the experimental treatment. This involves assessing efficacy. Evans et al. [34] argue that efficacy data should be provided at each interim analysis, even if no group-sequential procedure has been implemented. These authors argue that the DMC should get a complete picture of the benefit-risk profile of the experimental treatment before taking a decision. To compensate for the interim looks they suggest to “spend’’ a minuscule amount of the type 1 error, such as 0.0001, each time efficacy data are reviewed. However, it seems unlikely that this type of “sham” correction has any effect in practice. It appears to us that if the purpose of the efficacy look is to put the safety into perspective, and only the DMC — not the trial sponsor! — receives any efficacy information, then no correction for multiple testing should be applied. The question is then whether such efficacy data should be provided at all interim analyses, or only when an imbalance in safety occurs. The Bayesian approach offers an alternative way to perform interim efficacy looks, which are based on posterior probabilities. By default, no correction for multiple testing is implemented. Rather, the decision to stop for efficacy or futility is based on the amount of information on the efficacy expressed by the posterior probability that is available at the time of the interim analysis. Details of such a Bayesian approach can be found, e.g., in Lesaffre et al. [35] In any case, it is important to check with the regulatory authorities what is acceptable, and the plan should be clearly spelled out in the DMC charter.

Efficacy outputs, if provided, should be restricted to essential information on the primary and possibly key secondary endpoints. This can be a simple table. Of note, for some indications efficacy is measured by “lack of safety events,” such as absence of major cardiovascular events. In this case, similar types of displays can be used for efficacy as for safety, see, e.g., Evans et al. [34] Alternatively, one could use a figure like Fig. 2, corrected for the time at risk.

## Discussion

This article was motivated by the frustration of many DMC clinicians that one of the authors collaborated with over two decades. Consistently, the DMC members were asked to assess the safety of trial participants from a large volume of often poorly organized, difficult-to-read outputs, in little time. Improved DMC outputs and processes have been proposed in the literature, but they are rarely put into practice. The goal of this article was to increase awareness, synthetize existing proposals, and suggest possible solutions for better-serving DMCs. We started from a principled approach, clearly calling out the problem, and spelling out four main questions that a DMC is most interested in addressing. We proposed several effective plots as alternatives to commonly produced tables, and we provide related R code in the supplementary material. Of note, all our examples assume that the DMC report is fully unblinded, to provide the DMC with the most relevant information.

Our intention was to provide a useful piece to the puzzle of making DMCs more effective. The code provided will hopefully be useful to others. We refer to Buhr et al., [3] DeMets and Wittes [4], and Ellenberg et al., [36] for details on the logistical setup and organization of the various parties involved in the production and review of DMC reports. Importantly, the DMC can and should play an active role in the design of a DMC report. This requires an early involvement of the DMC, and it facilitates an early interaction between clinical and statistical experts on the DMC. The DMC process should also be set up such that potential additional ad-hoc requests by the DMC members can be quickly accommodated. We also see a competitive opportunity for contract research organizations (CROs) by offering services that improve and modernize DMC reports, and by providing support and training to DMC members, especially with interactive reporting options.

One may point out that a twenty-first century DMC report should be an interactive one, allowing to switch from overall tables and figures to individual data by pointing and clicking. However, most clinical researchers are not familiar with such dedicated software, which implies a steep learning curve to get familiar with the way relevant information can be extracted. We argue that such interactive software is most useful when used by the unblinded statistician at the time of the DMC meeting.

Some may object that the implementation of new outputs and processes for a DMC is inefficient, compared to simply taking a subset of the planned Clinical Study Report outputs. However, considering the risks, and the resource wasted by an inconvenienced DMC and additional one-off requests, this may not be true. Easing the reviewers’ task will save the sponsor’s resources. Also, the two situations are different. While a Clinical Study Report should summarize all study results after study completion, the DMC takes an active role during trial conduct and must be enabled to grasp key safety information very quickly — ancillary details only distract. We believe that, once better DMC processes and products are put in place by an initial effort, they can be re-used and will enhance overall efficiency in the long run. Safety domains are also more standardized than efficacy, which creates opportunities to standardize processes and packages, perhaps even across sponsors. Finally, from a different point of view, Clinical Study Reports may also benefit from some learnings that we gain by improving the effectiveness of DMC reports and products.

## Conclusions

In summary, rather than producing large volumes of material, we should design simple displays that answer the questions of interest and support the reviewers’ workflow. This applies not only, but also, to DMCs. We hope that this paper contributes to a change in practice, serving DMCs with more effective information packages, to enable them to perform their important task as well as possible.

## Supplementary Information

**Additional file 1.** Source outputs from the Xanomeline Clinical Study Report (Word doc). Program code in R: https://github.com/DMC21cent/DMC21cent.

## Data Availability

- Data: CDISC Pilot replication repository. - Code: https://github.com/DMC21cent/DMC21cent

## References

[1] European Medicines Agency (EMA). Committee for medicinal products for human use. Guideline on Data Monitoring Committees, 2006 https://www.ema.europa.eu/en/documents/scientific-guideline/guideline-data-monitoring-committees_en.pdf. 10.1002/sim.2585 16639775

[2] Food and Drug Administration (FDA). Guidance for Clinical Trial Sponsors: Establishment and Operation of Clinical Trial Data Monitoring Committees, 2006. https://www.fda.gov/media/75398/download

[3] Buhr KA, Downs M, Rhorer J, Bechhofer R, Wittes J (2018). Reports to independent data monitoring committees: an appeal for clarity, completeness, and comprehensibility. Ther Innov Regul Sci.

[4] DeMets D, Wittes J (2022). Data monitoring committee interim reports: we must get there soon!. Clin Trials.

[5] Wang W, Revis R, Nilsson M, Crowe B (2021). Clinical trial drug safety assessment with interactive visual analytics. Stat Biopharm Res.

[6] Zink RC, Wolfinger R, Mann G (2013). Summarizing the incidence of adverse events using volcano plots and time intervals. Clin Trials.

[7] Wildfire J, Bailey R, Krouse RZ, Childress S, Sikora B, Bryant N, Rosanbalm J, Wilson E, Modell JG (2018). The safety explorer suite: interactive safety monitoring for clinical trials. Ther Innov Regul Sci.

[8] O’Connell M, Pietzko K (2010). Interactive clinical data review for safety assessment and trial operations management.

[9] Evans SR, Zeng L, Dai W (2023). The data and safety monitoring board: the toughest job in clinical trials. N Engl J Med (NEJM) Evid.

[10] Fleming TR, DeMets DL, Roe MT, Wittes J, Calis KA, Vora AN, Meisel A, Bain RP, Konstam MA, Pencina MJ, Gordon DJ, Mahaffey KW, Hennekens CH, Neaton JD, Pearson GD, Andersson TL, Pfeffer MA, Ellenberg SS (2017). Data monitoring committees: promoting best practices to address emerging challenges. Clin Trials.

[11] Fleming TR, Ellenberg SS, DeMets DL (2018). Data monitoring committees: current issues. Clin Trials.

[12] Calis KA, Archdeacon P, Bain R, De DeMets M, Elzarrad MK, Forrest A, McEachern J, Pencina MJ, Perlmutter J, Lewis RJ (2017). Recommendations for data monitoring committees from the clinical trials transformation initiative. Clin Trials.

[13] Mütze T, Friede T (2020). Data monitoring committees for clinical trials evaluating treatments of COVID-19. Contemp Clin Trials.

[14] Thomas S, Jung K, Sun H, Psioda MA, Quibrera PM, Strakowski SM (2020). Enhancing clarity of clinical trial safety reports for data monitoring committees. J Biopharm Stat.

[15] Duke S, Bancken F, Crowe B, Soukup M, Botsis T, Forshee R (2015). Seeing is believing: good graphic design principles for medical research. Stat Med.

[16] Vandemeulebroecke M, Baillie M, Margolskee A, Magnusson B (2019). Effective visual communication for the quantitative scientist. CPT Pharmacometrics Syst Pharmacol.

[17] CTSpedia: https://www.ctspedia.org.

[18] R Development Core Team. R: a language and environment for statistical computing. (R Foundation for Statistical Computing, Vienna, Austria, 2008) <http://www.R-project.org>.

[19] CDISC Pilot replication repository, 2019: https://bitbucket.cdisc.org/projects/CED/repos/sdtm-adam-pilot-project/browse

[20] Amit O, Heiberger RM, Lane PW (2008). Graphical approaches to the analysis of safety data from clinical trials. Pharm Stat.

[21] Gordon I, Finch S (2015). Statistician heal thyself: have we lost the plot?. J Comput Graph Stat.

[22] Bush V (1945). As we may think. The Atlantic.

[23] Wang W, Nilsson M, Revis R, Crowe B (2020). Interactive visualization for clinical safety data review. Biopharm Rep 2019.

[24] Shneiderman B. The eyes have it: A task by data type taxonomy of information visualizations. Proc. IEEE Visual Languages 1996:336–343.

[25] Vandemeulebroecke M, Baillie M, Carr D, Kanitra L, Margolskee A, Wright A, Magnusson B (2019). How can we make better graphs? An initiative to increase the graphical expertise and productivity of quantitative scientists. Pharm Stat.

[26] Wisconsin DMC report, 2016. https://biostat.wiscweb.wisc.edu/research/clinical-trials/statistical-data-analysis-center/dmc-reports/

[27] Moher D, Hopewell S, Schulz KF, Montori V, Gøtzsche PC, Devereaux PJ, Elbourne D, Egger M, Altman DG, CONSORT 2010 (2010). CONSORT 2010 Explanation and Elaboration: updated guidelines for reporting parallel group randomised trials. Br Med J..

[28] Herson J (2017). Data and Safety Monitoring Committees in Clinical Trials.

[29] Harrell F, 2020. http://hbiostat.org/R/hreport/report.html#content

[30] Rho, 2021. https://rhoinc.github.io/safety-outlier-explorer/test-page/

[31] Rho, 2021. https://rhoinc.github.io/paneled-outlier-explorer/test-page/

[32] Powsner SM, Tufte ER (1994). Graphical summary of patient status. Lancet.

[33] patientProfilesVis, 2021. R package by Cougnault, L. https://cran.rstudio.com/web/packages/patientProfilesVis/

[34] Evans SR, Bigelow R, Chuang-Stein C, Ellenberg SS, Gallo P, He W, Jiang Q, Rockhold F (2020). Presenting risks and benefits: helping the data monitoring committee do its job. Ann Intern Med.

[35] Lesaffre E, Baio G, Boulanger B (2020). Bayesian Methods in Pharmaceutical Research.

[36] Ellenberg SS, Fleming TR, DeMets DL (2002). Data Monitoring Committees In Clinical Trials: A Practical Perspective.

