# Supplementary material for: DMC reports in the 21st century: towards better tools for decision-making
Source: Trials. 2023 Apr 21;24:289. doi: 10.1186/s13063-023-07290-4 (PMC10120491; doi:10.1186/s13063-023-07290-4)

**DMC reports in the 21^st^ century. Towards better tools for decision-making**

Marc Vandemeulebroecke, Mark Baillie, Ardalan Mirshani, Emmanuel Lesaffre

**Additional file 1:** **Source outputs from the Xanomeline Clinical Study Report**

**Supplementary Table 1: Demographics from the Xanomeline Clinical Study Report (excerpt)**


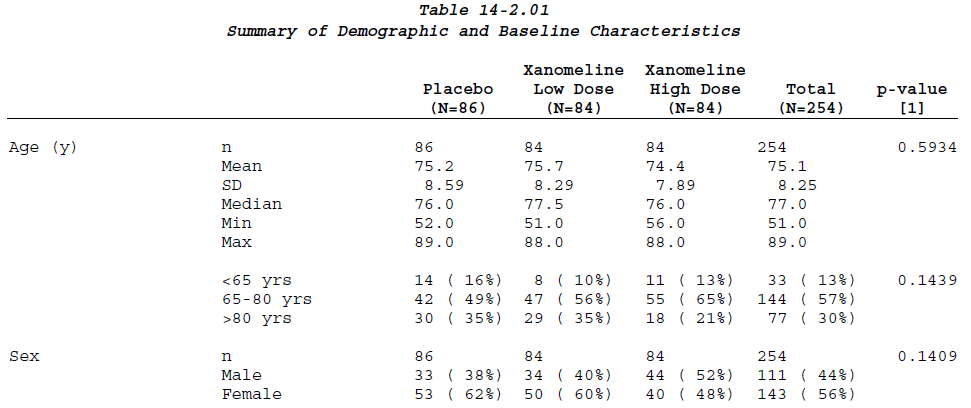


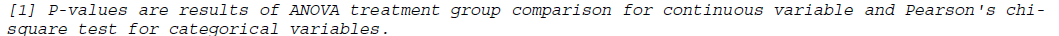


**Supplementary Table 2: Adverse events from the Xanomeline Clinical Study Report (excerpt)**


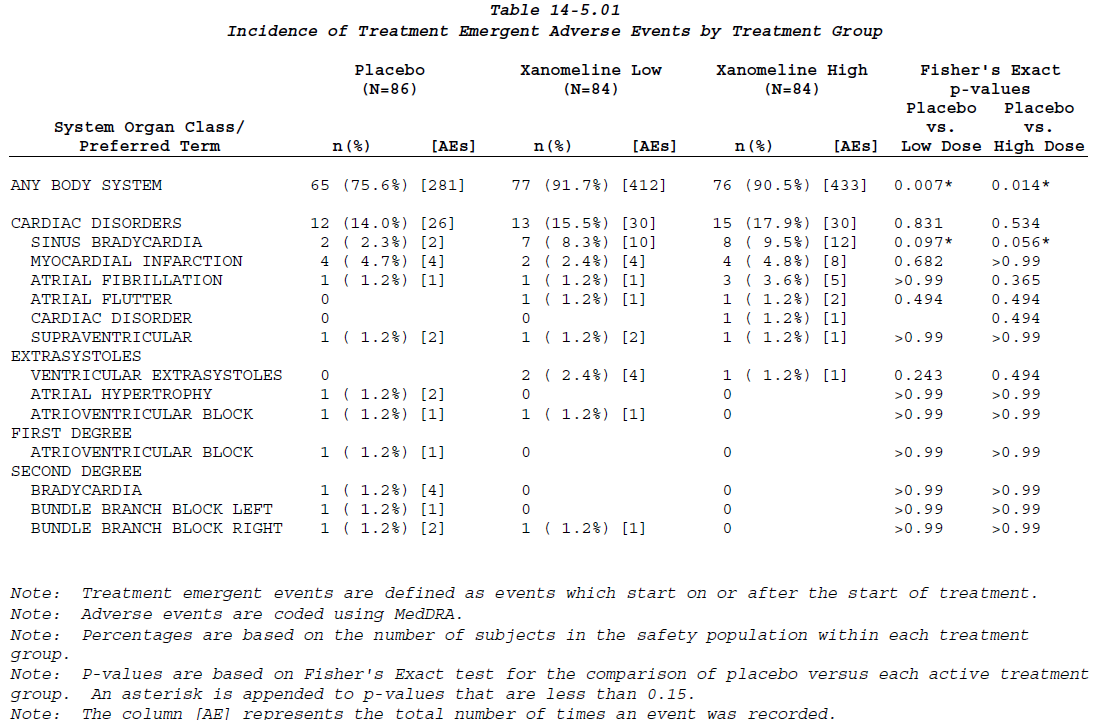


**Supplementary Table 3: Serious adverse events from the Xanomeline Clinical Study Report (excerpt)**


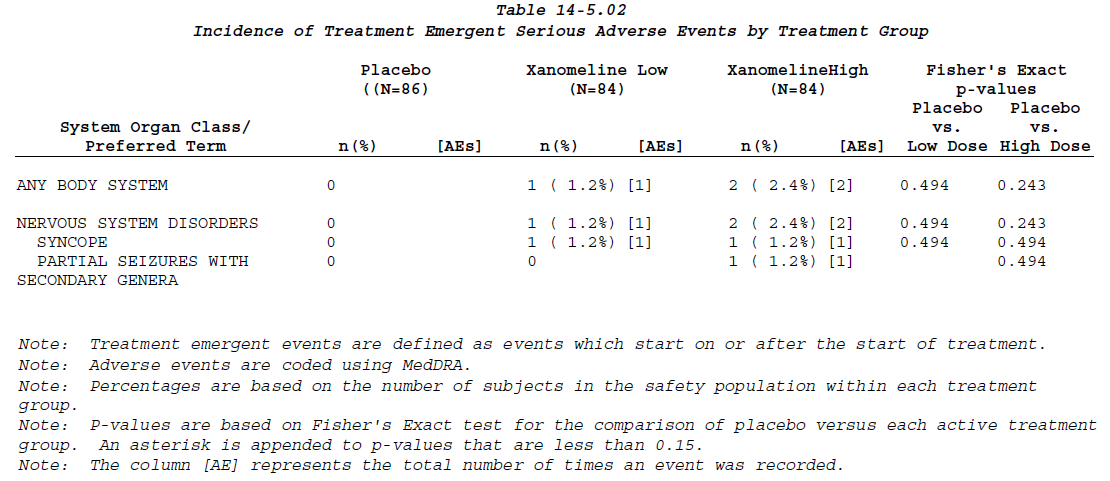


**Supplementary Table 4: Summary statistics of continuous laboratory parameters from the Xanomeline Clinical Study Report (excerpt)**


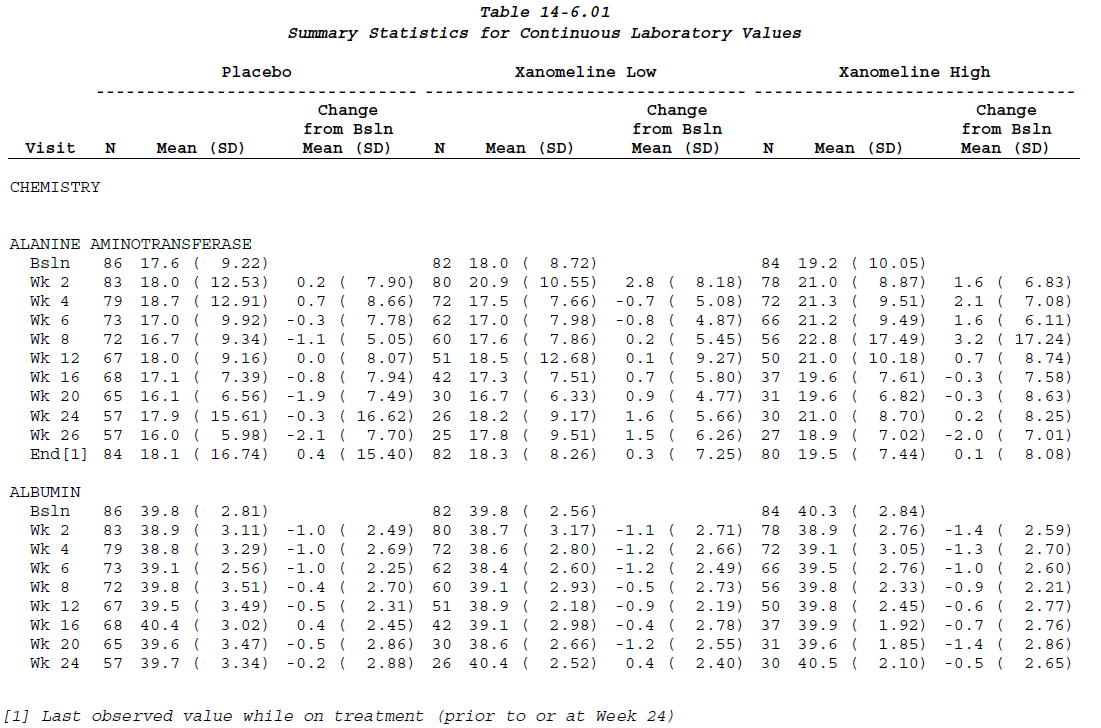


**Supplementary Table 5: Abnormal laboratory values from the Xanomeline Clinical Study Report (excerpt)**


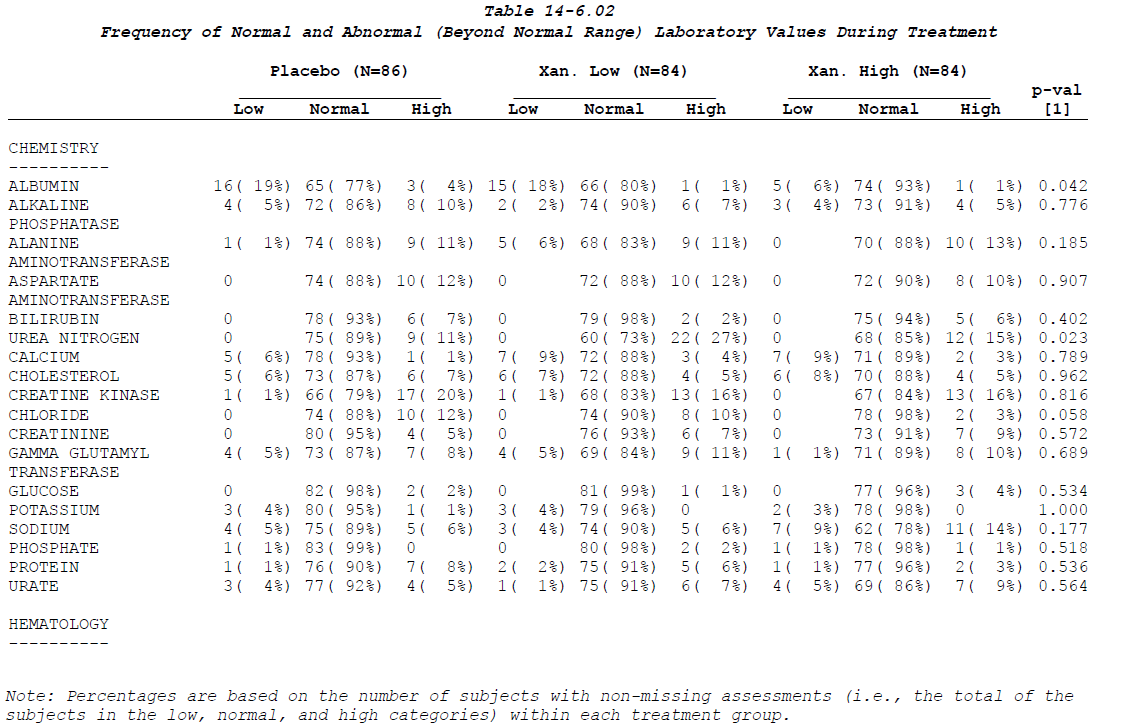


**Supplementary Table 6: Laboratory shift table from the Xanomeline Clinical Study Report (excerpt)**


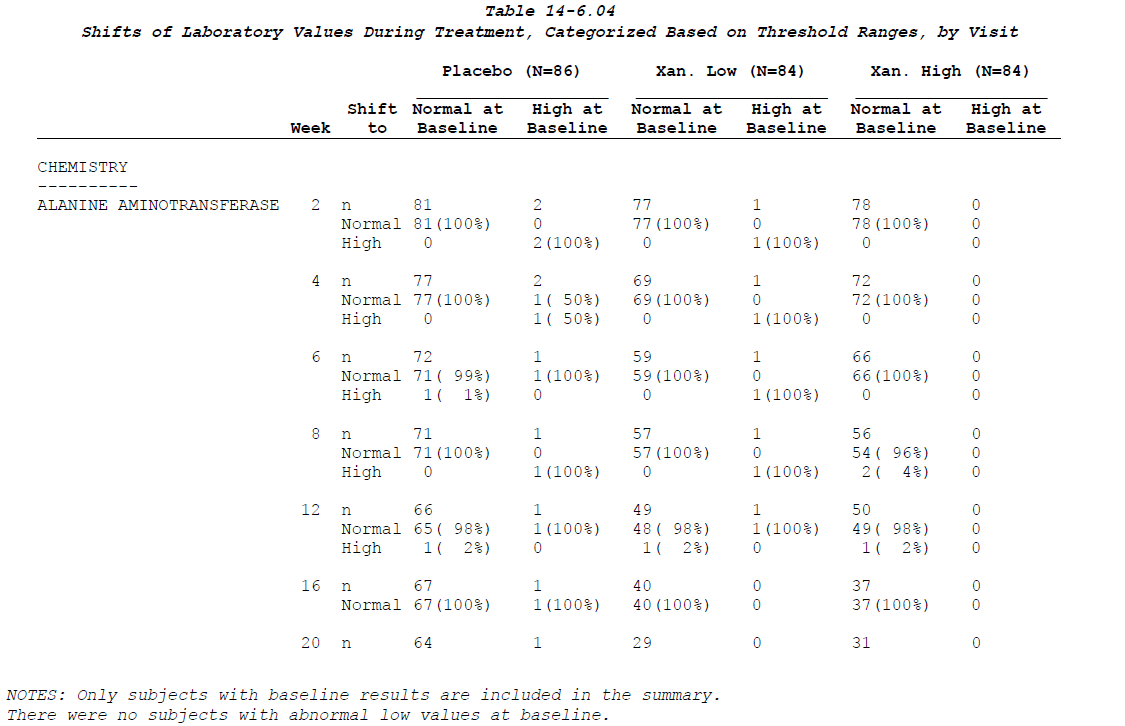

Supplement: Supplementary file 1 — Additional file 1. Source outputs from the Xanomeline Clinical Study Report (Word doc). Program code in R: https://github.com/DMC21cent/DMC21cent. [file 13063_2023_7290_MOESM1_ESM.docx]
